# Head and neck hemangiopericytoma in a child: case report

**DOI:** 10.1590/S1516-31802004000500010

**Published:** 2004-09-01

**Authors:** Jomar Rezende Carvalho, Leonardo Haddad, Fernando Danelon Leonhardt, Marcílio Ferreira Marques, Rodrigo de Oliveira Santos, Onivaldo Cervantes, Márcio Abrahão

**Keywords:** Hemangiopericytoma, Head and neck neoplasms, Vascular tissue neoplasms, Paranasal sinus, Soft tissue neoplasms, Hemangiopericitoma, Neoplasias de tecido vascular, Neoplasias de cabeça e pescoço, Seios paranasais, Neoplasias de tecido mole

## Abstract

**CONTEXT:**

Hemangiopericytoma is a relatively rare tumor, first described in 1942, with approximately 300 cases described in the literature to date. In most cases, it affects the trunk and lower extremities. The head and neck incidence is less than 20%, mostly in adults. We describe a case of malignant head and neck hemangiopericytoma in a child.

**TYPE OF STUDY:**

Case report.

**CASE REPORT:**

A twelve-year-old male patient noted the presence of a firm painless right-side retroauricular lymph node of 1 cm in diameter, which at first remained unchanged for six months, but subsequently enlarged progressively. He denied having had previous trauma at that site. In November 2000, he presented nasal obstruction and voluminous epistaxis that required hospitalization and blood transfusion. During dental treatment one month later, a cranial x-ray revealed bone alterations. A subsequent computed tomography scan showed an extensive lesion of soft tissue density that had invaded the maxillary fossa, eroding the skull base and middle and nasal fossa. The child was then referred to our service, where biopsy was performed, giving a diagnosis of hemangiopericytoma. Shortly afterwards, magnetic resonance imaging revealed that this lesion had undergone significant growth, while maintaining the same invasion pattern. The patient was submitted to conservative surgery in April 2001, with only partial resection of the tumor because of its extent. Histopathological examination of the specimen confirmed the presence of malignant hemangiopericytoma. Following the surgery, the patient presented fast regrowth of the lesion, with partial response to chemotherapy and radiotherapy.

## RESUMO

**CONTEXTO:**

O hemangiopericitoma é uma neoplasia relativamente, descrita pela primeira vez em 1942, e com aproximadamente 300 casos descritos até hoje na literatura. Na maioria dos pacientes, acomete o tronco e membros inferiores, sendo a incidência em cabeça e pescoço menor que 20%, e principalmente em adultos. Relatamos um caso de hemangiopericitoma de cabeça e pescoço em criança.

**TIPO DE ESTUDO:**

Relato de caso.

**RELATO DO CASO:**

Paciente do sexo masculino, 12 anos, que, em dezembro de 1999, notou nódulo retroauricular à direita, endurecido, indolor e de 1 cm de diâmetro, que evoluiu estável por seis meses e com posterior crescimento progressivo. Negava história de trauma. Em novembro de 2000, apresentou obstrução nasal e epistaxe volumosa, necessitando de internação e transfusão sangüínea. Um mês depois, durante tratamento dentário, uma radiografia craniana evidenciou alterações ósseas. Tomografia computadorizada subseqüente mostrou lesão extensa característica de partes moles, invadindo fossa maxilares, destruindo base do crânio, fossa média e fossas nasais. A criança foi encaminhada ao nosso serviço, onde biópsia foi realizada, com diagnóstico de hemangiopericitoma. Logo após exame de ressonância nuclear magnética evidenciou a mesma lesão com aumento importante, mas invadindo as mesmas estruturas. Submetido à cirurgia conservadora em abril de 2001 com ressecção parcial do tumor devido à sua extensão. O exame histopatológico revelou hemangiopericitoma maligno. Após a cirurgia, o paciente apresentou rápido crescimento da massa tumoral e resposta parcial a quimioterapia e radioterapia.

## INTRODUCTION

Hemangiopericytoma is a rare tumor type, first described in 1942 by Stout and Murray. It originates in the pericytes, a specific cell type identified by Rouget in 1873 and subsequently described by Zimmermann in 1923. These are cells with smooth muscle characteristics that are highly arborized and arranged alongside capillary vessels. They have a contractile capability and are responsible for vessel caliber regulation, modulating both flux and permeability.^1-3^

Hemangiopericytoma accounts for about 1% of all vascular tumors. About 5% of such cases occur in the nasal cavity and usually consist of well-differentiated tumors with low potential for local recurrence or metastasis. Hemangiopericytoma usually evolves with slow and non-painful growth that progresses towards nasal obstruction and epistaxis, which are the most common symptoms.^1,4^ When this tumor only invades the nasal fossa and paranasal sinus, the prognosis is more favorable than in the case of meningeal or other patterns of invasion. The treatment of choice is surgical resection. Regarding etiology, a past history of trauma, prolonged steroid use and hypertension are said to have some correlation, but such correlations have not been formally demonstrated.^5^ We describe here a case of head and neck hemangiopericytoma in a child with none of the above predisposing factors.

## CASE REPORT

In December 1999, a white twelve-year-old boy noted the presence of a firm painless right-side retroauricular node of 1 cm in diameter. It was treated as lymphadenitis, with no alteration for six months, after which it exhibited progressive enlargement. In November 2000, the child presented nasal obstruction and massive epistaxis, requiring blood transfusion.

**Figure 1:**
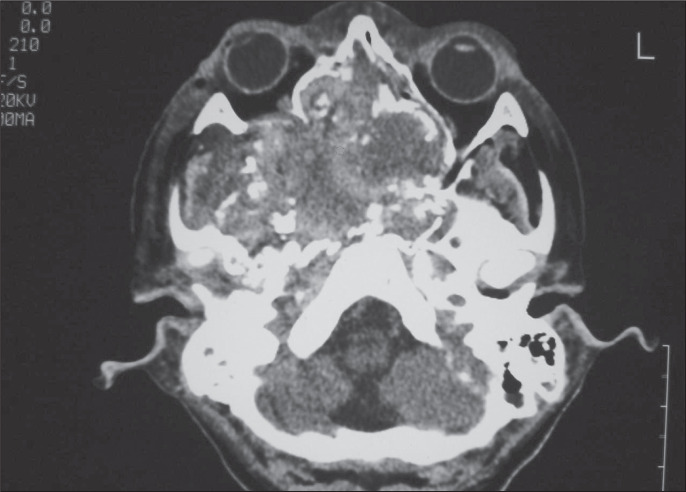
Computed tomography scan of a twelve-year-old patient, showing lesion of soft tissue density invading maxillary fossa, eroding the skull and middle and nasal fossa.

In December 2000, during dental treatment, a cranial x-ray revealed some bone alterations, and the patient was referred to a physician. A cranial computed tomography scan was requested (Figure 1), which showed an extensive lesion of soft tissue density that had invaded the maxillary fossa, eroding the skull base and middle and nasal fossa.

**Figure 2:**
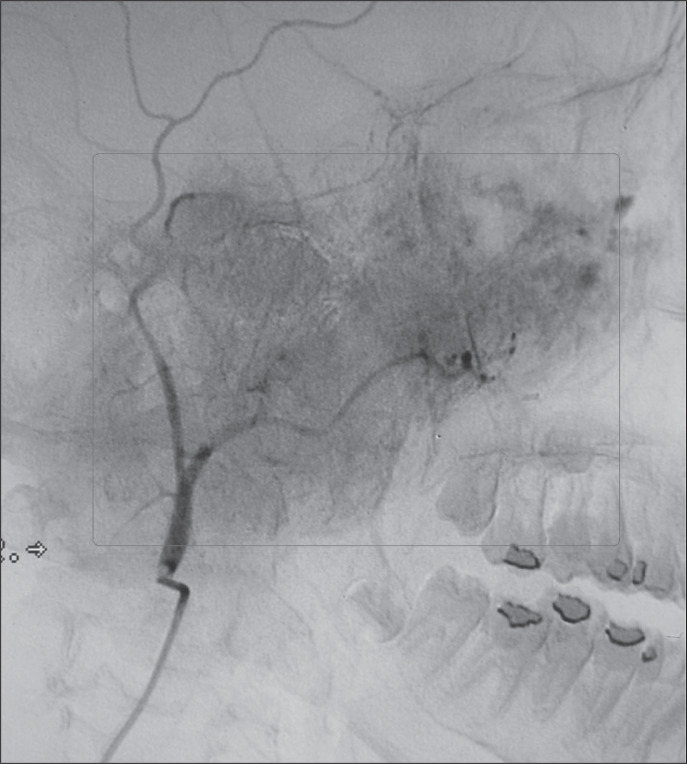
Magnectic resonance imaging of a twelve-year-old patient, showing solid mass with isodense contrast in T1.

**Figure 3:**
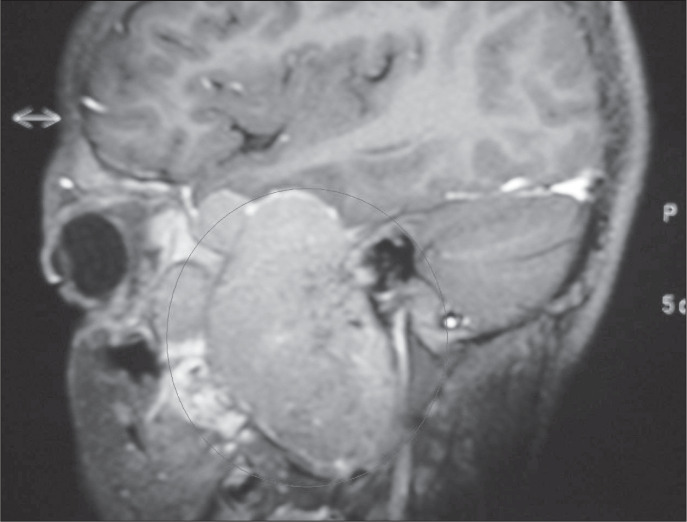
Angiography of a twelve-year-old patient.

The child was then brought to our service, where biopsy was performed, giving a diagnosis of hemangiopericytoma. Magnetic resonance imaging (Figure 2) and angiography (Figure 3) performed in March 2001 revealed that this lesion had undergone significant growth, while maintaining the same invasion pattern as shown by the computed tomography scan.

He again presented epistaxis in April 2001, and emergency surgery was performed to control the hemorrhaging. The surgical approach involved a Weber-Ferguson incision (lateral rhinotomy with upper lip splitting and extending to the lower eyelid) with en bloc excision of the medial maxilla and the ethmoid labyrinth. Radical resection was not possible because of the massive extension of the tumor into the skull base. Histopathological examination of the specimen confirmed the presence of malignant hemangiopericytoma.

Following surgery, because of the presence of a liquor fistula, he was referred first to chemotherapy, receiving four cycles of vincristine, actinomycin and cyclophosphamide and one of iphosphamide and vepeside. Subsequently, he received further irradiation via linear accelerator because of endocranial infiltration. The tumor responded to the last irradiation, with local control of the disease, and the patient remains asymptomatic until the present date.

## DISCUSSION

Apart from being rare in absolute terms, hemangiopericytoma is uncommon in the head and neck. Stout and Murray (1942) described 691 cases of vascular tumors, and only nine of these were hemangiopericytoma.^6^ Since then, approximately 300 cases of hemangiopericytoma have been described, mostly in the trunk or lower extremities. Its incidence in the head and neck is less than 20%.^6,7^ In this site, it mostly affects soft tissue around the oral cavity, sinus tract and meninges; and more rarely, it affects the orbit, parotid gland, skull base and temporal bone.^4,6-8^

Its peak prevalence is in the sixth to seventh decade of life: it is rare among adolescents or children. The individual's sex does not influence its incidence.^1,7^ Our patient had no past history of trauma, hypertension or prolonged steroid use.

Diagnosis of highly vascularized tumors in the head and neck is challenging, especially because of the difficulty in differentiating hemangiopericytoma from other tumors that have prominent vascularization: juvenile hemangioma, glomus tumor, angiosarcoma, leiomyoma, leiomyosarcoma, schwannoma, mesothelioma, liposarcoma, benign and malign histiocytoma, synovial sarcoma, chondrosarcoma, neuroblastoma, adenoid cystic carcinoma and mixed cell tumor.^1,4,5^ Angiographic features may help in differentiating hemangiopericytoma from other hypervascular lesions (Figure 3). Tomography, radiography and angiography are not specific and magnetic resonance imaging reveals a solid mass with isodense contrast in T1.

Cell pleomorphism and mitotic activity are uncommon in hemangiopericytoma of the paranasal sinuses. Enzinger reported the following characteristics that are compatible with a high-grade tumor: nuclear atypia, necrosis, hemangioma, presence of more than four mitosis per microscope field and size greater than 6.5 cm.^6,7^ However, Stout and Murray^3^ did not notice any correlation between the mitosis level and tumor behavior. They noted that the ten-year survival rates of patients with lesions that presented fewer than four mitosis per microscope field, absence of necrosis and size below 6.5 cm were respectively 77%, 81% and 92%. On the other hand, when the tumor presented more than four mitosis per field, necrosis and size greater than 6.5 cm, the ten-year survival rates were respectively 9%, 29% and 63%.^7^

Recurrence indicates a poor prognosis and many such cases develop distant metastasis. Studies have shown metastasis rates ranging from 18% to 69%.^7,8^ Patients with hemangiopericytoma should be regularly monitored for local recurrence and systemic tumor spread.

The treatment of choice is total local excision. Adjuvant radiotherapy and chemotherapy may be employed and, although the literature is not quite clear about their results, recent studies have suggested that their use is indicated mostly in cases where only partial resection was performed, as in our patient.
